# C-reactive protien levels among Indian patients with acute ischemic stroke

**DOI:** 10.6026/9732063002001413

**Published:** 2024-10-31

**Authors:** Nityanand Billa, Anjali More, Vasant Deokar

**Affiliations:** 1 Krishna Institute of Medical Sciences, Karad, Maharashtra, India

**Keywords:** C-reactive protein, stroke incidence, Indians, severity, outcomes, acute ischemic stroke, cerebral function disturbances

## Abstract

Level of C-reactive protein among Indian patients with acute ischemic stroke is of interest in the management of myocardial infarction. Hence, C-reactive protein levels among 50 Indian patients with acute ischemic stroke were measured. Data shows significant difference with below and above levels of C-reactive protein among stroke estimated myocardial infarction patients. Thus, incorporating C-reactive protein level assessments into routine diagnostic test is important for the evaluation of stroke.

## Background:

Cerebral blood volume, accounts for the majority, ranging between 60-80% of stroke cases, whereas hemorrhagic stroke stems from vascular rupture, leading to intracranial bleeding [1]. Classification of hemorrhagic stroke according to studies, includes Intracerebral hemorrhagic and subarachnoid hemorrhagic, each presenting unique challenges in management and prognosis [1-2]. Stroke is the second leading cause of death mortality and the third leading cause of long-term disability, inflicting a significant socioeconomic burden [3]. 68% of all strokes worldwide are ischemic with hemorrhagic accounting for the remaining 32% [4]. Data indicates a significant prevalence of ischemic stroke, which represents 87% of cases in USA. Hemorrhagic stroke follows at 10%, while subarachnoid hemorrhagic accounts for approximately 3% [3]. Gender disparities in the prevalence of sexually transmitted infections and incidence of disease are evident, with women exhibiting significantly higher age-adjusted rates compared to men. This finding indicates a noteworthy public health concern [5]. Acute coronary syndrome is highly prevalent in the Indian demographic, ranking among the highest worldwide [6]. Even with the reestablishment of blood circulation, neurons within the penumbra face significant threats to their viability, particularly from excitotoxicity and inflammation [7]. Studies have also shown that neuronal depolarization or cell death causes the neurotransmitter glutamate to be released without control. This leads to excitotoxicity, which is a major cause of neuronal damage in ischemic stroke [8]. Once this disturbance occurs, it triggers the activation of the apoptotic cascade, mitochondrial malfunction, the production of reactive oxygen and nitrogen species, and the activation of (adenosine diphosphate-ribose) polymerase [3]. An acute-phase reactant called C-reactive protein can rise dramatically, up to 1,000 fold, at sites of infection or inflammation [4]. Therefore, it is of interest to investigate the role of C-reactive protein as a biomarker for risk assessment, prognosis and treatment response for acute ischemic cases.

## Materials & Methods:

The current single-center, hospital-based, prospective, observational analytical study was conducted over a period of 18 months from November 2022 to April 2024 with sample size of 50 patients. C-reactive protein levels were measured and recorded at the time of admission and detailed clinical evaluations and necessary investigations were performed to assess the neurological status and confirm the diagnosis of acute ischemic stroke.

## Inclusion criteria:

[1] stroke patient

[2] Developing clinical signs of focal or global (coma) NL deficit which lasted for more than 24 hours or leading to death, with no other apparent cause than vascular origin.

[3] Patients that presented within 48 hours of onset of stroke.

## Exclusion criteria:

Patients with sub-arachnoid hemorrhagic, extra-dural hemorrhagic, sub-dural hemorrhagic and intra-cerebral hemorrhagic based on CT scan results of the brain were excluded.

## Statistical analysis: 

Data was analyzed using SPSS version 25.0. Independent t-tests and analysis of variance were employed to compare mean C-reactive protein levels between two or more groups, respectively. Pearson correlation coefficients assessed the relationship between C-reactive protein levels and National Institutes of Health Stroke Scale scores.

## Results:

Table 1 shows that the average age was seen up to 69.1 years and standard deviation with 11.58 years. Whereas, male dominancy was seen with 26 in number (52%) followed by female with 24 in number (48%) respectively. Table 2 shows that 12% C-reactive protein levels below 7 mg/L, while 88% had levels exceeding 7 mg/L. Table 3 shows that no significant difference in age between participants with C-reactive protein levels below 7 mg/L (mean age 70.17 years, SD 15.70) and those with C-reactive protein levels of 7 mg/L or higher (mean age 68.95 years, SD 11.13), as the p-value was 0.81. Table 4 shows that among females, 33.33% had C-reactive protein levels below 7 mg/L & 50.00% had levels of 7 mg/L or higher. Among males, 66.67% had C-reactive protein levels below 7 mg/L and 50.00% had levels of 7 mg/L or higher. We found non-significant difference as the p value was 0.44. Table 5 shows that C-reactive protein levels below 7 mg/L had a mean weight of 69.17 kg (SD = 11.10), while those with C-reactive protein levels of 7 mg/L or higher had a mean weight of 71.79 kg (SD = 15.34). We found non-significant difference as the p value was 0.68. Table 6 shows that C-reactive protein levels below 7 mg/L had lower National Institutes of Health Stroke Scale scores initially (15.33 ±10.27) and after 5 days (13.83 ±8.97) compared to those with C-reactive protein levels of 7 mg/L or higher, who had higher scores both initially (25.45 ±10.01) and after 5 days (25.45 ±10.07). We found statistically significant difference as the p value was 0.02 and 0.01 respectively. Table 7 shows that hypercholesterolemia 100.0% had C-reactive protein levels below 7 mg/L, while 68.18% of them had levels of 7 mg/L or higher.

None of the participants without hypercholesterolemia had C-reactive protein levels below 7 mg/L, and 31.82% had levels of 7 mg/L or higher. We found non-significant difference as the p value was 0.10. Table 8 shows that none of the participants with a history of MI had C-reactive protein levels below 7 mg/L, and 34.09% had levels of 7 mg/L or higher. We found non-significant difference as the p value was 0.09. Table 9 shows that significant difference in Systolic blood pressure between participants with C-reactive protein levels below 7 mg/L (mean 146.67 mmHg) and those with levels of 7 mg/L or higher (mean 160.22 mmHg). We found significant difference as the p value was 0.007. However, there was no significant difference found for D-BP as the p value was 0.54 respectively. Table 10 shows that 0 patients had C-reactive protein levels (<7) while 10 patients found C-reactive protein < 7 group. We found non-significant difference as the p value was 0.19. Table 11 shows that only 1 patient (16.67%) had C-reactive protein levels below 7, while a substantial (79.55%) 35 patients had C-reactive protein levels of 7 or higher. We found highly significant difference as the p value was 0.001 respectively. Table 12 shows that only 1 patient (16.67%) showed C-reactive protein levels below 7, while 36 patients (81.82%) had C-reactive protein levels of 7 or higher. We found highly statistically significant difference as the p value was 0.0007. Table 13 shows that 2 patients (33.33%) had C-reactive protein levels below 7, while 36 patients (81.82%) had C-reactive protein levels of 7 or higher.

We found significant difference as the p value was 0.009. Table 14 shows that among individuals with stroke, 12.00% had C-reactive protein levels below 7, while a significant majority of 88.00% had C-reactive protein levels of 7 or higher. In contrast, among those without stoke, 68.00% had C-reactive protein levels below 7, with 32.00% having C-reactive protein levels of 7 or higher. We found highly significant association as the p value was <0.0001 respectively.

## Discussion:

Our study investigates the levels of C-reactive protein in patients with acute ischemic stroke cerebrovascular accident and their association with various demographic, clinical and treatment-related factors. The average age of the study participants was 69.1 years with a standard deviation of 11.58 years. The gender distribution was balanced, with 48% females and 52% males. This demographic data aligns with the typical age range for stroke patients, indicating that the sample is representative of the broader population affected by acute ischemic stroke. This finding highlights the role of inflammation, as indicated by elevated C-reactive protein levels, in acute ischemic stroke. Men had greater average serum C-reactive protein levels than women did, according to Almeida *et al.* [9]. The distribution of these C-reactive protein serum levels did vary by age and sex, however. The C-reactive protein serum levels and were found to be much lower in men aged 75, whereas in women it was reverse. Lakoski *et al.* women had substantially higher median C-reactive protein levels compared with men (2.56 vs 1.43 mg/L, P < .0001) [10]. According to Khera *et al.* women had higher C-reactive protein levels than men (median, 3.3 vs. 1.8 mg/l; p < 0.001) [11]. Straatman *et al.* showed no effect was observed for gender, age, and BMI on postoperative C-reactive protein levels. The mean weight of patients with C-reactive protein levels less than 7 mg/L was 69.17 kg, compared to 71.79 kg in those with higher C-reactive protein levels. The p-value of 0.68 suggests no significant difference in weight between the two groups, implying that the weight of the patient does not significantly affect C-reactive protein levels in acute ischemic stroke patients [12]. Analysis of clinical parameters revealed significant associations between elevated C-reactive protein levels and higher National Institutes of Health Stroke Scale scores both on admission and after 5 days, reflecting more severe strokes among patients with higher C-reactive protein levels. This finding underscores the potential of C-reactive protein as a prognostic marker for stroke severity and functional outcome. Elevated levels of C-reactive protein might be a reflection of the extent of brain injury. Patients with C-reactive protein levels 7 mg/L had better NIHSS scores on admission; according to a study by Hertog *et al.* [13] Patients with higher C-reactive protein levels have larger infarctions, according to older studies. In addition to indicating the degree of tissue damage, C-reactive protein may also point to a condition of higher risk brought on by higher inflammation or an excess of cytokines [14, 15]. Recent experimental studies revealed that C-reactive protein itself may cause additional damage to the brain after localized cerebral ischemia, potentially via a complement-mediated aggravation of tissue injury [16]. The difference was not statistically significant, despite the fact that a larger proportion of hypercholesterolemia patients had increased C-reactive protein levels.

Pradhan *et al.* showed patients with type 2 diabetes mellitus had higher C-reactive protein levels compared to non-diabetic patients [17]. Another nested case-control study of 550 middle-aged women followed for four years found that those in the top quartile of the C-reactive protein distribution had a nearly 16-fold higher chance of acquiring type 2 diabetes than those in the bottom quartile. Although the correction for BMI and other variables reduced the connection, it remained substantial and significant, with a relative risk of 4.2 [18]. In our study, significant association was observed between smoke/tobacco use and elevated C-reactive protein levels (p = 0.009). This indicates that smokers and tobacco users are more likely to have higher C-reactive protein levels, suggesting that smoking contributes to inflammation in acute ischemic stroke [19]. Moreover, Tonstad *et al.* found that C-reactive protein levels were significantly higher in both male and female smokers compared with non-smokers (median values of 1.0 mg/l and 11.2 mg/l for male non-smokers and smokers, respectively, and for females 2.0 mg/l and 11.6 mg/l, respectively). This highlights the substantial impact of smoking on systemic inflammation, as evidenced by elevated C-reactive protein levels [20]. Elevated levels of C-reactive protein are associated with more severe stroke, higher Systolic blood pressure, and worse functional outcomes [21]. It has been shown that high levels of high-sensitivity C-reactive protein are present in all subtypes of ischemic stroke and that these levels are independently related to atherosclerosis of the large arteries and cardio-embolic stroke. A threefold increase in the risk of acquiring a cardio-embolic stroke and a twofold increase in the risk of developing atherosclerosis in the large arteries were related to high levels of high-sensitivity C-reactive protein in stroke subtypes. Because of this, higher levels of high-sensitivity C-reactive protein in these subtypes might be a sign that primary prevention efforts should start. Because of this, high levels of high-sensitivity C-reactive protein may be a sign that it's time to start taking statins for both primary and secondary prevention. To investigate these results, further studies on a large scale are necessary in the future [22]. It has a sensitivity of 80% and a specificity of 75%. A C-reactive protein level of 10.25 mg/L is linked to a severe ischemic stroke. Additionally, this level correlates with an unfavorable stroke outcome as measured by the modified Rankin Scale, exhibiting a specificity of 75% and an outcome prediction of 82%. The C-reactive protein level did not correlate with the severity of illness or the outcomes of stroke scores in cases of hemorrhagic stroke. It is feasible to predict the severity and early outcome of an ischemic stroke based on the serum C-reactive protein level at the time of admission; however, this predictive capability does not extend to hemorrhagic stroke [23]. In addition to the widely acknowledged disorders, elevated C-reactive protein levels represent an independent risk factor [24].

## Conclusions:

Data shows statistically significant associations between elevated C-reactive protein levels and the presence of arterial hypertension, diabetes mellitus and smoke/tobacco use. This implies that C-reactive protein is a valuable indication of inflammation and stroke severity. Thus, these variations must be taken into account in clinical evaluations.

[1] Small sample size

[2] Single centered study could limit the generalized finding

[3] Study not conducted long term study

[4] The inclusion criteria required patients to present within 48 hours of stroke onset and give informed consent. This might exclude patients who present later or are unable to consent, potentially introducing selection bias.

[5] C-reactive protein levels were measured at specific time points.

[6] Variation in the timing of C-reactive protein measurements relative to stroke onset could affect the results, as C-reactive protein levels can fluctuate significantly post-stroke.

[7] Although the study adjusted for confounding like age, sex, and comorbidities, there might be other variables that are unmeasured could influence the results.

[8] Different treatment protocols, lifestyle factors, and genetic predispositions were not controlled for in the study.

## Figures and Tables

**Table 1:** DMG distribution

**Age (Mean ± SD)**	**69.1 ± 11.58**	
**Gender**	**Total**	**Percentage**
Female	24	48.00%
Male	26	52.00%
Total	50	100.00%

**Table 2:** According to CRP level

**CRP level (N=50)**	**CRP< 7**		**CRP> 7**	
	**No. of cases**	**Percentage**	**No. of cases**	**Percentage**
No of cases	6	12.00%	44	88.00%

**Table 3:** Age wise distribution

**Age**	**CRP<7**	**CRP≥ 7**	**t-test**	**p-value**
(Mean ± SD)	70.17 ± 15.70	68.95 ± 11.13	-0.24	0.81

**Table 4:** Gender wise distribution

**Gender**	**CRP< 7**	**CRP≥ 7**	**Chi square**	**p-value**
	**No of cases (%)**	**No of cases (%)**		
Female	2(33.33%)	22(50.00%)	0.57	0.44
Male	4(66.67%)	22(50.00%)		
Total	6(100.00%)	44(100.00%)		

**Table 5:** Distribution of weight

**Weight**	**CRP< 7**	**CRP≥ 7**	**t-test**	**P-value**
(Mean ± SD)	69.17 ± 11.10	71.79 ± 15.34	0.4	0.68

**Table 6:** NIHSS type

**NIHSS**	**CRP< 7 (Mean ± SD)**	**CRP≥ 7 (Mean ± SD)**	**P-value**
NIHSS on admission	15.33 ± 10.27	25.45 ± 10.01	0.02
NIHSS after 5 days	13.83 ± 8.97	25.45 ± 10.07	0.01

**Table 7:** HCST

**Hypercholesterolemia (HCST)**	**CRP< 7**	**CRP≥ 7**	**Chi square**	**P-value**
	**No of cases (%)**	**No of cases (%)**		
No	0(0.00%)	14(31.82%)	2.59	0.1
YES	6(100.00%)	30(68.18%)		
Total	6(100.00%)	44(100.00%)		

**Table 8:** MI distribution

**Myocardial infarction (MI)**	**CRP< 7**	**CRP≥ 7**	**Chi square**	**P-value**
	**No of cases (%)**	**No of cases (%)**		
NO	6(100.00%)	29(65.91%)	2.86	0.09
Yes	0(0.00%)	15(34.09%)		
Total	6(100.00)	44(100.00%)		

**Table 9:** Physical examination distribution

**Physical examination**	**CRP< 7**	**CRP≥ 7**	**t-test**	**P-value**
Systolic blood pressure (S-BP)	146.67 ± 8.16	160.22 ± 11.51	2.78	0.007
Diastolic blood pressure (D-BP)	91.67 ± 7.52	94.09 ± 9.23	0.61	0.54

**Table 10:** Death distribution

**Death**	**CRP< 7**	**CRP≥ 7**	**Chi-square**	**P-value**
	**No of cases (%)**	**No of cases (%)**		
NO	6(100.00%)	34(77.27%)	1.67	0.19
Yes	0(0.00%)	10(22.73%)		
Total	6(100.00%)	44(100.00%)		

**Table 11:** Arterial hypertension (at-hy-t)

**Arterial hypertension**	**CRP< 7**		**CRP≥ 7**		**Chi square**	**P-value**
**Case**	**No. of case**	**Percentage**	**No. of case**	**Percentage**		
NO	5	83.33%	9	20.45%	10.14	0.001
YES	1	16.67%	35	79.55%		
Total	6	100.00%	44	100.00%		

**Table 12:** Diabetes mellitus distribution

**Diabetes mellitus**	**CRP< 7**		**CRP≥ 7**		**Chi square**	**P-value**
**Case**	**No. of case**	**Percentage**	**No. of case**	**Percentage**		
NO	5	83.33%	8	18.18%	11.41	0.0007
YES	1	16.67%	36	81.82%		
Total	6	100.00%	44	100.00%		

**Table 13:** SMK/ tobacco consumption

**Smoker / Tobacco consumption**	**CRP< 7**		**CRP≥ 7**		**Chi square**	**P-value**
**Case**	**No. of case**	**Percentage**	**No. of case**	**Percentage**		
NO	4	66.67%	8	18.18%	6.66	0.009
YES	2	33.33%	36	81.82%		
Total	6	100.00%	44	100.00%		

**Table 14:** ST type

**Type of stroke**	**CRP< 7**		**CRP≥ 7**			**Chi square**	**P-value**
	**No. of case**	**Percentage**	**No. of case**	**Percentage**	**Total**		
Stroke	6	12.00%	44	88.00%	50	32.67	< 0.0001
Non stroke	34	68.00%	16	32.00%	50		
Total	40		60		100		
